# Hepatitis E Virus Assembly and Release

**DOI:** 10.3390/v11060539

**Published:** 2019-06-09

**Authors:** Xiaohui Ju, Qiang Ding

**Affiliations:** Center for Infectious Diseases Research, School of Medicine, Tsinghua University, Beijing 100084, China; juxh15@mails.tsinghua.edu.cn

**Keywords:** hepatitis E virus, life cycle, assembly, release

## Abstract

Hepatitis E is an underestimated threat to public health, caused by the hepatitis E virus (HEV). HEV is the most common cause of acute viral hepatitis in the world, with no available direct-acting antiviral treatment. According to a recent WHO report, 20 million people become infected with HEV annually, resulting in 44,000 deaths. However, due to the scarcity of efficient in vitro cell culture systems for HEV, our knowledge of the life cycle of HEV is incomplete. Recently, significant progress has been made towards gaining a more comprehensive view of the HEV life cycle, as several in vitro culturing systems have been developed in recent years. Here, we review current knowledge and recent advances with regard to the HEV life cycle, with a particular focus on the assembly and release of viral particles. We also discuss the knowledge gaps in HEV assembly and release. Meanwhile, we highlight experimental platforms that could potentially be utilized to fill these gaps. Lastly, we offer perspectives on the future of research into HEV virology and its interaction with host cells.

## 1. Introduction

Hepatitis E virus (HEV) remains a considerable health problem in developing and developed countries. Globally, there are approximately 20 million HEV infections each year, leading to 3.3 million symptomatic cases and 44,000 related deaths [1,2]. HEV infections are usually self-limiting in healthy individuals. However, HEV can establish chronic infections and induce cirrhosis in immunosuppressed patients, such as organ-transplant patients and patients infected with human immunodeficiency virus (HIV) [3,4,5,6,7]. The overall fatality rate of HEV infection in the general population is about 1%. However, in pregnant women, it can be a serious illness with a high mortality rate of up to 30% during the third trimester of pregnancy [8,9]. The mechanisms underlying this very severe pathogenesis remain incompletely understood. Originally, HEV infection was considered to be a disease limited to developing countries with poor sanitation [10], but HEV is now increasingly recognized as an infection that is also prevalent in developed countries. In the latter case, HEV is primarily transmitted through the consumption of infected animal meat products [11,12,13,14].

HEV is a member of the *Hepeviridae* family of the *Orthohepevirus* genus, which can be further subdivided into four species (A, B, C, and D). HEV strains infecting humans include genotypes 1, 2, 3, 4, and 7, which fall under the species *Orthohepevirus A* [15]. Swine is considered as the major reservoir for zoonotic HEV infection [16,17]. Recently, it was reported that rat HEV of the *Orthohepevirus C* species was able to infect a patient who was a liver transplant recipient [18]. While a prophylactic vaccine to prevent HEV infection has been developed and is licensed in China, it is not yet available elsewhere. In addition, direct-acting antivirals are not yet available in clinical practice. Thus far, chronic hepatitis E is treated with ribavirin and pegylated interferon (PEG-IFN), however, this therapy is associated with numerous side effects, and since ribavirin is teratogenic, its use is counter-indicated in pregnant women.

Various HEV cell culture systems have been developed [19], however, the life cycle of HEV is still not well characterized. The entry process of HEV is poorly understood and the host receptor has not yet been identified. Moreover, the replication process and the essential host factors for HEV replication remain elusive. In this review, we summarize the recent advances in HEV virology and highlight the gaps in our knowledge on the late stages of the HEV life cycle, specifically on the assembly and release of infectious HEV particles. We sincerely apologize to our colleagues whose important contributions could not be cited due to the limited space and scope of this review.

## 2. HEV Genome Organization and Encoded Proteins

HEV possesses a positive-sense single-stranded RNA genome of approximately 7.2 kb in length, which is flanked by 5’ and 3’ untranslated regions (UTR). Both 5’UTR and 3’UTR contain important *cis*-acting elements required for HEV replication, translation, or assembly. There is a 7-methylguanine cap at the 5’ end and a poly(A) tail at the 3’ end, which resembles host mRNA [20,21]. The HEV genome contains three open reading frames (ORFs), while a fourth ORF is exclusively identified in HEV-1 (genotype 1) and has been shown to interact with multiple viral and host proteins to enhance virus replication [22]. ORF1 encodes a large nonstructural protein including multiple functional domains, such as methyltransferase, putative papain-like cysteine protease (PCP), X domain, helicase, and RNA-dependent RNA polymerase (RdRp), which is responsible for HEV genome replication [23]. ORF1 can also replicate the HEV genome in trans by its overexpression in HepG2/C3A cells [24]. However, whether ORF1 needs to be cleaved into individual sub-units based on the functional domains is still controversial [25,26,27,28,29]. Both ORF2 and ORF3 products are translated by a subgenomic RNA with a length of 2.2 kb, which is transcribed from the negative-sense genomic RNA [30]. ORF2 protein is the capsid protein, which encapsulates the viral genome into viral particles and interacts with the putative host receptors. Moreover, the viral capsid formed by the ORF2 antigen is also a major target for neutralizing antibodies. The licensed vaccine containing amino acid residues 368 to 606 of the ORF2 from HEV-1 has been proven to be effective against HEV infection [31]. The ORF3 protein is a 13 kDa phosphorylated protein with multiple functions [32,33,34,35,36,37]. Evidence suggests that ORF3 is an ion channel important for HEV egress [38]. In this review, we will focus on the late stages of the HEV life cycle, including the assembly (capsid assembly and genome encapsidation) and release (quasi-enveloped virion formation and release from infected cells) of virions.

## 3. Assembly: Viral Genomic RNA Encapsidation and Virion Formation

ORF2 is a HEV capsid protein and can package the HEV genome into viral particles. Previously, ORF2 has been divided into two major functional domains: The shell (S) domain and the protrusion (P) domain, just like the Norwalk virus [39]. The S domain includes N-terminal residues folding into eight anti-parallel β barrels to form the virus shell. The P domain consisting of C-terminal residues is engaged in dimeric contact to form the protrusions [40]. Recently, it has been found that ORF2 contains three domains, S (shell), M (middle), and P (protruding) [41], and S, the continuous capsid, contains P1, 3-fold protrusions, and P2, 2-fold spikes [42]. While the persistent infection system of HEV has been established [19], sufficient amounts of viral particles cannot be obtained for the study of its structure. Therefore, many efforts have been made to produce empty virus-like particles (VLPs) by overexpressing ORF2 proteins in vitro [41,42,43,44,45]. Evidence suggests that the truncated form (amino acid residues 112 to 660) of ORF2 can generate a VLP which is a *T* = 1 icosahedral particle composed of 60 copies of the truncated products of the capsid protein [44,45]. The crystal structure shows that the truncated ORF2s (amino acids 112–608) of both genotype-3 and genotype-4 form *T* = 1 VLPs [41,42]. However, genotype-3 ORF2 protein, after deleting 52 residues from the C terminus, can successfully produce a *T* = 3 virion-sized VLP which contains nucleic acid [46]. The N-terminal basic region or the genomic RNA plays an important role in switching off the transition from *T* = 1 to *T* = 3 symmetry [41,42,46]. It has been suggested that the dimeric ORF2 proteins first assemble into a pentamer, then into an icosahedral capsid. The pentamer of dimers may be the assembly intermediate, which is common for the *T* = 3 virus [46,47]. The diameter of the formed *T* = 1 VLP is 23.7 nm, which is a little smaller than the 27 nm of native HEV particles collected from infected monkeys [43]. The crystal structure of the dimerization domain of the recombinant capsid protein (ORF2 amino acid residues 455–602) shows that this domain forms a tight homodimer and the neutralizing antibody recognition site is located in this domain [48].

**Figure 1:**
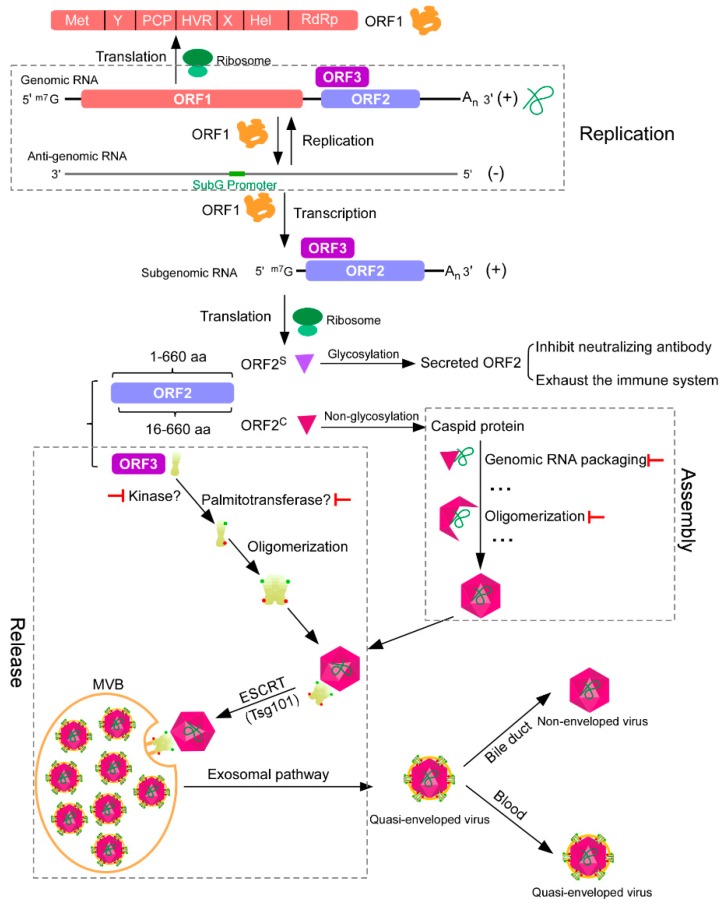
Schematic representation of hepatitis E virus (HEV) replication, assembly, and release. After HEV enters the cell, the ORF1 is translated by the host ribosome. The ORF1 can replicate HEV genomic RNAs and transcribe subgenomic RNAs. Subgenomic RNAs encode both ORF2 and ORF3. ORF2 is a capsid protein, which has two forms, secreted and capsid, translated from different start codons. Secreted ORF2 can function as a decoy to inhibit the neutralizing antibody or to exhaust the immune system. Capsid ORF2 can bind genomic RNA and oligomerize to form viral particles. ORF3, which can be phosphorylated and palmitoylated to form oligomer, is required for viral particle release from host cells, functioning as an ion channel. ORF3 can interact with both ORF2 and tumor susceptibility gene 101 (Tsg101), a component of the cellular endosomal sorting complexes required for transport (ESCRT) machinery. In this way, the viral particle is transported by multivesicular bodies (MVBs) through the exosomal pathway, which then fuse with the plasma membrane to release quasi-enveloped viruses out of cells. Quasi-enveloped viruses released from the apical domain of the hepatocytes enter the bile duct, where the lipid envelope is degraded by detergents in the bile, while viruses released from the basolateral domain enter the blood in their quasi-enveloped form. 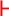 represents the potential antiviral target.

It is believed that viral genomic RNA encapsidation is mediated by ORF2 interaction with HEV genomic RNA, which brings the virus genome into the capsid and forms the infectious particles. ORF2 evolves a mechanism to distinguish the viral genomic RNA from the host RNA at the site for assembly to ensure a functional virion package. The N terminus of ORF2 contains a signal peptide, which is followed by an arginine-rich domain, and this region is potentially involved in RNA encapsidation during virus assembly [49,50]. Approximately 110 amino acid residues (aa) of the N-terminal region of ORF2 specifically interact with the 76-nt region (encapsidation signal) in the 5’ end of the HEV genome [50]. However, a more detailed genetic analysis is required to identify the encapsidation signal and the essential amino acids of ORF2 needed to mediate such interaction. HEV is postulated to transcribe subgenomic RNA transcripts encoding ORF2 and ORF3 [30]. Hence, it is conceivable that the RNA encapsidation signal is localized at the 5’ end of the genome, which ensures that only the full-length genomic RNA is recognized and packaged by the capsid protein. It is thought that the ORF2 will oligomerize to form an icosahedral capsid, within which the genomic RNA is encapsidated, and the oligomerization is triggered after ORF2 binds to the encapsidation signal (Figure 1). However, the mechanism of genomic RNA-induced ORF2 oligomerization and the domain of ORF2 responsible for this oligomerization are still unknown. 

Due to a scarcity of robust cell culture models, the assembly of HEV particles is still poorly understood. There are some fundamental questions regarding the assembly: (1) What is the place of the assembly? The initiation of virion assembly is thought to require the release of replicated genomes to allow contact with the ORF2 protein, which forms the virion capsid. Currently, little is known about the site for HEV virion assembly. Lipid droplets (LDs) are cellular lipid storage organelles which maintain lipid homeostasis [51]. Previous studies found that hepatitis C virus (HCV) and dengue virus (DENV) infections could induce the accumulation of LDs, and that a core protein localized in the LDs is responsible for the virion assembly [52,53,54]. To better understand HEV virion assembly, confocal and electron microscopy should be employed to understand the subcellular localization of ORF2 and to identify the potential viral assembly site. (2) What is the mechanism to switch from replication to assembly? Is it possible that ORF1 is responsible for such a switch? RNA modifications, especially *N*6-methyladenosine (m6A), can regulate RNA structure, localization, stability, and function. The m6A modification on the HCV RNA genome has been reported to influence the interaction of HCV RNA with its core protein [55]. Whether the m6A modification on the HEV RNA genome can regulate this switch needs to be determined. (3) Do any host factors participate in the viral particle assembly? It is conceivable that the assembly of HEV virions is dependent on numerous host factors, which are not yet known. Combined with proteomic and genetic approaches, we could identify the host factors involved in viral particle assembly, which will not only further our understanding of HEV assembly, but also explore more attractive drug targets for therapeutics. (4) Are any host proteins associated with viral particles? It has been reported that a number of host proteins are associated with HCV virions, such as nucleoporin Nup98 [56] and apolipoprotein E (apoE) [57]. The biochemical and biophysical properties of HEV virions remain incompletely characterized due to the limited availability of experimental models capable of robustly supporting virion morphogenesis and the release of infectious particles. The characterization of the composition of HEV virions will facilitate our understanding of the host contribution to HEV propagation and pathogenesis. The APOE gene encodes the apoE protein, which combines with lipids in the body to form lipoproteins. There are three apoE protein isoforms (apoE2, apoE3, and apoE4), differing from each other by either one or two amino acids at positions 112 and 158, respectively [58]. These isoforms are encoded by three allelic variants of APOE: e2, e3, and e4, respectively. Intriguingly, it was reported that the ApoE protein was upregulated during acute HEV infection in a swine model, as demonstrated by quantitative proteomics analysis [59,60]. Meanwhile, a serological, Third National Health and Nutrition Examination Survey (NHANES III)-based, cross-sectional study suggested that APOEe3 and e4 may be associated with protection against HEV infection in non-Hispanic blacks but not in non-Hispanic whites or Mexican Americans [58]. Conversely, Steinmann and colleagues did not successfully confirm the functions of APOEe3 and e4 in HEV replication, assembly, and release in an in vitro cell culture model [61]. Therefore, these controversial results suggest that the ApoE protein probably plays some role in HEV infection in an allele-dependent manner in vivo, but this requires further investigation. Genetic analysis in vivo is urgently needed in an appropriate animal model to clarify the exact role of ApoE single nucleotide polymorphisms (SNPs) or other potential factors involved in HEV susceptibility.

## 4. HEV Particle Release

### 4.1. Infectious Viral Particle Release

After the assembly of the viral particles, progeny virions will be released to initiate another round of infection. ORF3 is a phosphoprotein of 113 or 114 amino acids. The ORF3 protein is not required for virus replication, assembly or entry into hepatoma cells in vitro [62]; it dictates virus egress or release from infected cells [63], however the mechanism is not well characterized. Based on previous studies, we can briefly outline the process of viral particle release as follows (Figure 1): (1) The targeting of the assembled virions. Phosphorylated ORF3 can interact with the non-glycosylated ORF2 protein [64]. ORF3 is not required for virion assembly; it is conceivable that ORF3 interaction with non-glycosylated ORF2 may be the mechanism for ORF3 to recognize the viral particles for release; (2) The trafficking of the virion to the multivesicular bodies (MVBs) through endosomal sorting complexes required for transport (ESCRT) machinery. ORF3 interacts not only with ORF2 proteins, but also with numerous host proteins, such as microtubules in the cytoskeleton [32] and tumor susceptibility gene 101 (Tsg101), a component in the ESCRT machinery [65,66]. ORF3 can interact with the microtubules through its N-terminal hydrophobic domains, leading to the elevation of acetylated α-tubulin, which increases microtubule stability [65]. The physiological relevance of this interaction to the HEV life cycle is not elucidated; it probably enables the virions to employ the microtubules for intracellular transport until the ESCRT machinery is available. It is suggested that MVBs of the late endosomal compartment mediate the release of HEV particles. MVB biogenesis relies on the ESCRT protein complexes [67,68]. Mechanistic studies found that a proline-rich motif containing PSAP amino acids in the ORF3 C-terminal could interact with Tsg101 to exploit the ESCRT machinery in order to load the virions into the MVBs for release [66]. Moreover, since cargo proteins engaging the MVBs’ sorting machinery are tagged with ubiquitin as the sorting signal [69,70], the sorting mechanism for loading the HEV virion into MVBs is not well characterized. (3) Envelopment with the host membranes (quasi-enveloped HEV). HEV sheds into the feces as non-enveloped virions (naked virions), but circulates in the blood as a membrane-associated, quasi-enveloped form (eHEV) [71,72,73]. The majority of cell-culture-derived HEV particles are in eHEV form [74]. The eHEV particles have lipid membranes on their surface, which is associated with ORF3 but not observed in non-enveloped particles [72,75]. The host-derived membrane of eHEV masks the ORF2 antigen to comprise efficacy of neutralizing antibodies. Subsequent studies have demonstrated that the naked virion and eHEV utilize distinct mechanisms to enter into the hepatocytes [72]. Nagashima and his colleagues purified the endosome and analyzed eHEV composition using immunoprecipitation and real-time RT-PCR methods, revealing that eHEV particles released by the cellular exosomal pathway are copurified with exosomes in the exosome fraction. Tetraspanins (CD63, CD9, and CD81), epithelial cellular adhesion molecule (EpCAM), and phosphatidylserine (PS), as well as trans Golgi network protein 2 (TGOLN2), are present on the surfaces of eHEV virions, indicating that membrane components are common among eHEV particles and exosomes. Additionally, electron microscopy image analysis of the purified eHEV suggests that the capsids of eHEV particles are individually covered by lipid membranes that resemble the lipid membranes of exosomes, similar to enveloped viruses [76,77]. However, the biogenesis of eHEV remains incompletely understood and we still do not have a comprehensive and unambiguous characterization of the composition of the host membranes surrounding eHEV. To better address this question, an unbiased, quantitative proteomics analysis of eHEV could supply more insights into eHEV biogenesis and the subcellular origin of the host membrane, which will facilitate our understanding of HEV interaction with the host and HEV release. (4) The release of virions from cells. The MVBs containing eHEV fuse with plasma membranes to release eHEV, a process regulated by Rab27, as the depletion of Rab27 decreased the viral particles released from cells [78,79]. Moreover, CD63 and CD81, the ubiquitous markers of exosomes, have been found to associate with quasi-enveloped HEV virions, further suggesting that eHEV hijacks the secretory exosomes derived from MVBs for egress [79]. Meanwhile, ORF3 could oligomerize and function as an ion channel; its ion channel activity is also required for viral particle release. Of note, the PSAP motif is not necessary for ORF3’s ion channel activity [38]. The mechanism of the ion channel activity required for HEV release is not clear, although it probably involves adjusting the ion concentration and pH of the endosome to establish a conductive environment for virion release. Besides phosphorylation, ORF3 was found to be palmitoylated at cysteine residues in its N-terminal region by a yet unidentified palmitoyltransferase, which not only determines its membrane association and subcellular localization, but is also required for infectious particle release from cells [80]. It is conceivable that the post-translational modifications (phosphorylation, palmitoylation and other unknown modifications) on ORF3 constitute a dynamic process, which probably determines the different ORF3 subcellular localizations and thus forms ORF3 as a multifunctional protein. 

Hepatocytes are polarized epithelial cells in vivo, which have a uniquely organized polarity with distinct apical (facing the bile canaliculi) and basolateral (facing the hepatic sinusoid) domains in physiological conditions [81]. The progeny virions can release at both the apical and basolateral membranes of infected hepatocytes. Studies have found that ORF3 is mainly located close to the bile canaliculi of hepatocytes in vitro [82,83] and in vivo [84]. Most infectious HEV particles (as eHEV form) are released from the hepatocyte via its apical domain into the bile canaliculi, where they enter the biliary tract and are subsequently shed into feces, while a small fraction of HEV particles (as eHEV form) are released from the basolateral domain into the blood, where they can spread throughout the host. eHEV released from the apical domain enters the bile, and the eHEV membrane is degraded by the detergent action of bile, resulting in non-enveloped HEV in feces [71].

### 4.2. Secreted ORF2 Release

Recently, it has been reported that there are different forms of ORF2. A study reported that three forms of the ORF2 capsid protein have been characterized, namely, infectious/intracellular ORF2 (ORF2i), glycosylated ORF2 (ORF2g), and cleaved ORF2 (ORF2c). The ORF2i protein is an authentic capsid protein as a component of infectious HEV particles, whereas the ORF2g and ORF2c proteins are massively secreted glycoproteins which are not associated with infectious HEV particles [85]. Another study found that the translation of secreted ORF2 (ORF2^S^) is initiated at the previously presumed AUG start codon for the capsid protein, whereas the production of the actual capsid protein (ORF2^C^) is started at a previously unrecognized internal AUG codon (15 codons downstream of the first AUG). ORF2^S^, instead of ORF2^C^, exists as a glycosylated dimer and is efficiently released through the classic secretory pathway. Secreted ORF2 exhibits substantial antigenic overlap with the capsid, but they are not identical. [86]. Most expressed ORF2 is secreted out of cells as a secreted form instead of packaging the virus RNA genome to assemble viral particles. The role of secreted ORF2 in the HEV life cycle is unknown; it may function as a decoy to allow viral particles to escape from neutralizing antibodies. The conformation of ORF2^S^ and ORF2^C^ is not exactly the same, which implies that the host immune response against excessive ORF2^S^ generated during the natural HEV infection may not specifically target the ORF2^C^ antigen, and probably exhausts or delays the host immune system’s establishment of an effective protection against HEV infection. It will be worthwhile to analyze the secreted ORF2 level in chronic HEV infection cases. 

## 5. Viral Assembly and Release as Potential Antiviral Targets

Thus far, there is no direct-acting antiviral against HEV infection. The development of inhibitors which block the assembly or release of HEV particles would be an attractive research direction.

ORF2 plays crucial roles in HEV assembly, including genomic RNA packaging and folding into viral particles. It is not surprising therefore that ORF2 has been the target of attempts to develop antiviral drugs. An interesting target related to HEV assembly is the genomic RNA binding of ORF2. The RNA elements in the 5′ end of the genomic RNA can potentially be targeted to prevent its interactions with ORF2. Compounds or peptides specifically disrupting viral RNA binding to ORF2 could be developed to inhibit viral particle assembly. The oligomerization of ORF2 forms an icosahedral capsid, within which genomic RNA is encapsidated, which represents another promising target. Compounds that block or misdirect capsid assembly, leading to the suppression of virion production, could be used to treat hepatitis E as well. Usually, viral particle assembly is dependent on numerous cellular factors or pathways, which also serve as antiviral targets. However, no cellular factors or pathways have yet been identified as being involved in HEV assembly. Viral particle release is mediated by ORF3 protein. ORF3 links the assembled viral particles to the ESCRT pathway, which is mediated by ORF3 binding to the ESCRT component Tsg101 [66]. Therefore, the blockade of ORF3 binding to Tsg101, or the ESCRT pathway from cells, produces powerful blocks to HEV particle release. However, due to the important physiological function of the ESCRT pathway [87], the blockade of the ESCRT pathway is not an appropriate strategy for controlling HEV. Phosphorylation or palmitoylation of ORF3 is essential for viral particle release [38,80]; the identification of the host enzymes responsible for these modifications will supply more therapeutic options. Meanwhile, ORF3 ion channel activity is required for its function to release viral particles, providing an attractive potential target for direct-acting antiviral drug development [38]. The M2 proton channel of the influenza A virus is the target of the anti-influenza drugs amantadine and rimantadine, which have been used in clinics [88]. The identification of ORF3 ion channel inhibitors appears to be an area worthy of further work in the future.

It has been found that the interferon-induced gene (ISG) BST-2 (also known as CD317, HM1.24, or tetherin) holds fully formed HIV-1 virions to the cell surface, preventing their release [89,90]. However, its antiviral activity can be counteracted by the HIV-1 accessory protein Vpu [91]. BST-2 is a type II transmembrane protein (the N terminus is in the cytoplasm), and it is found at the plasma membrane and within several endosomal membrane compartments, including ESCRT, the trans-Golgi network (TGN), as well as early and recycling endosomes [92,93]. Given that eHEV particles exist in the cells, egress through ESCRT and exhibit resistance to neutralizing antibodies [74,79], BST-2 could potentially restrict HEV release. It will be worthy to test BST-2 for antiviral activity against HEV release.

## 6. Concluding Remarks and Future Perspectives

While the ability to recapitulate the entire HEV life cycle in cell culture has allowed us to gain initial insights in recent years toward understanding HEV assembly and release, many aspects of these processes still remain unclear. For example, what is the detailed mechanism of reciprocal interaction between viral factors and host factors for assembly and release? What is the exact mechanism of capsid and genomic RNA interaction to assemble viral particles, and where does this take place? How does eHEV acquire the host-derived membrane and what is the biochemical composition of the infectious HEV particle? Structural studies will be critical to clarify the exact composition of the eHEV virion. What is the precise role of ORF3 in the release process and why is ion channel activity required? Obviously, the existing cell culture model undoubtedly has limitations, and many of the above questions can only be addressed by developing more sophisticated cell culture models. For instance, the cells used to produce infectious viruses are derived from a hepatocellular carcinoma and do not possess many of the characteristics typical of hepatocytes in vivo. Recently, a number of new cell models that more readily reflect the properties of hepatocytes in vivo were developed [19,94,95]; what is of note is that a long-term 3D organoid culture system for human primary hepatocytes was established [96], which is an invaluable tool for HEV study. Additionally, an experimental system that can produce large amounts of highly purified infectious HEV particles is urgently required for structural investigations. While these are challenging tasks, given the rapid progress made in recent years, we can remain optimistic that the mystery of HEV assembly and release might be solved in the foreseeable future.
